# An Experimental Study on Torsional Behavior of Reinforced Concrete Columns Retrofitted with Hybrid Concrete Jackets

**DOI:** 10.3390/ma16031256

**Published:** 2023-02-01

**Authors:** Kyong Min Ro, Min Sook Kim, Young Hak Lee

**Affiliations:** Department of Architectural Engineering, Kyung Hee University, Deogyeong-Daero 1732, Yongin 17104, Republic of Korea

**Keywords:** seismic retrofitting, concrete jacketing, torsion, combined loading

## Abstract

The concrete jacketing method for retrofitting old reinforced concrete (RC) columns should secure confinement and seismic performance under torsion as well as unidirectional later loads. In a previous study, a hybrid concrete jacketing method was proposed using steel wire mesh (SWM), steel grid reinforcement (SGR), which can replace reinforcement of existing concrete jacketing method, and using steel fiber non-shrinkage mortar (SFNM). These details can simplify the retrofitting process of the existing concrete jacketing method, and seismic performance was evaluated by conducting a cyclic loading test under unidirectional loading. In this paper, the torsional behavior of RC columns retrofitted with the hybrid concrete jacketing method was investigated. Four specimens were fabricated and conducted cyclic loading tests under two types of loading schemes, unidirectional and bidirectional loading, to examine the effect of the loading path. The strength and energy dissipation capacity of retrofitted columns with hybrid concrete jackets increased approximately eight times compared to the old RC columns under torsional loading. Therefore, the hybrid concrete jacketing method can improve torsional resistance.

## 1. Introduction

Irregular buildings in which the rigidity or strength of a structural system is distributed discontinuously, horizontally, and vertically can experience seismic torsion due to discontinuity in the shape of the building and loading path. Many studies focused on the torsional behavior of reinforced concrete (RC) columns under combined load [1,2,3]. Tirasit and Kawashima [1] presented an experimental investigation on the behavior of RC columns subjected to combined cyclic loading, and experimental results showed that the flexural capacity decreases and the damage tend to occur upward outside the flexural plastic hinge region as the torsion increases. When the torsional load is applied to the RC column, significant shear cracks occur at the bottom of the column, and as the torsional load increases, concrete spalling is observed [1,2,3]. Therefore, torsional effects should be considered when attempting to improve seismic performance. Concrete jacketing is one of the most typical seismic retrofitting methods suggested by the U.S. Federal Emergency Management Agency [4] and has been proven to enhance strength and stiffness [5,6,7,8]. To secure confinement and seismic performance under torsion, interface treatment, which can increase bonding stress and the arrangement of reinforcing steel bars, should be significantly considered. In addition, to achieve adequate seismic performance under a combination of flexural, shear, and torsional loadings, the ductility of the concrete jackets must be improved. Fiber-reinforced polymer (FRC) can be used to handle this issue. Meda et al. [9] and Reggia et al. [10] retrofitted RC columns with concrete jackets using high-performance fiber(HPFRC). Meda et al. [9] reported that HPFRC could be greatly increased and the maximum story drift ratio and the crack control performance effectively improved, while strength cannot be improved as much as the expected performance. Reggia et al. [10] confirmed that the section enlargement is limited and additional reinforcement is typically required because of the lack of attachment performance and confinement strength of reinforced concrete columns. Gholampour et al. [11] conducted an axial compressive test of concrete columns retrofitted and confined by FRC jackets, and the test results showed that the fiber content affects the ductility and strength of columns using an FRC jacket. The steel fiber can also affect shear crack control under torsional load. Torsion failure is mainly due to the inherent weakness of concrete in tensile strength. The steel fibers can be significantly enhanced tensile properties and reduce the brittleness of concrete. Concrete members reinforced with steel fiber can arrest the opening and widening of micro-cracks due to the capability of steel fibers [12,13,14].

**Figure 1:**
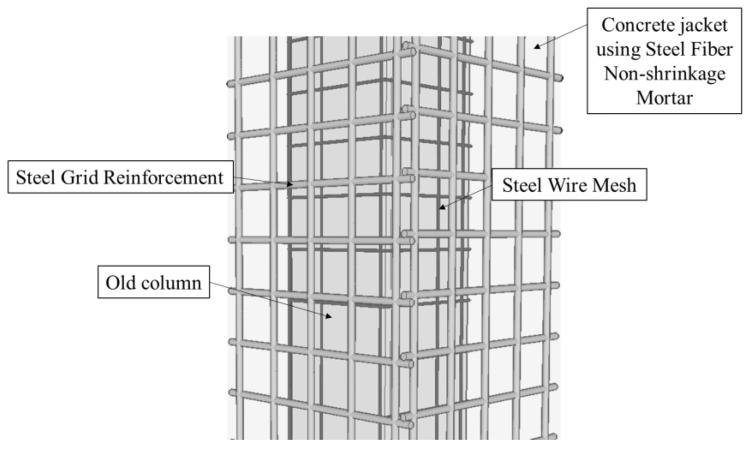
Hybrid concrete jacket [15].

To improve the seismic performance and ensure easy construction of the existing concrete jacketing retrofit method without interface treatment, a hybrid concrete jacketing method was proposed to strengthen the non-seismic RC columns [15]. The hybrid concrete jackets using steel wire mesh (SWM), steel grid reinforcement (SGR), and steel fiber non-shrinkage mortar (SFNM) are presented in Figure 1. The SWM is square in shape with narrow openings, which are formed by welding wires to ensure bonding between the old column and the concrete jacketing section. SGR is consisted of vertically and transversely arranged bars that can replace longitudinal bars and stirrups in jacketing section. These details can simplify the interface treatment and easily arrange reinforcement in jacketing section. The steel fiber was used to control shear cracks under torsional load. The previous study [15] conducted a cyclic loading test to evaluate the structural performance of reinforced concrete columns retrofitted with the proposed details. The experimental results showed that the proposed concrete jacketing method effectively increased the strength, initial stiffness, and energy dissipation compared with the old columns, which had a non-seismic design.

This study was focused on the evaluation of the torsional behavior of the hybrid concrete jacketing method under the combined load of compression, including a bending and torsional moment. The experimental results were used to verify the seismic performance and confinement effect of the hybrid concrete jacketing method in torsional loading.

## 2. Experimental Program

### 2.1. Specimen Details

Four reinforced concrete columns, including two old columns (NSU, NSB) and two retrofitted columns (SFU, SFB), were fabricated. The loading scheme used variables to investigate the effect loading path. Old columns with non-seismic details had 250 mm × 250 mm cross sections and were 1800 mm tall. The concrete jacketing section was designed according to Pennelis and Kappos [16]. The recommendation on the design concrete jacketing section was as follows [10]:

(1) The strength of the new concrete must be greater than that of the old concrete.
(2) The minimum thickness of the jacket should be 100 mm on each side, and four longitudinal reinforcements should be arranged at least.
(3) The spacing of the stirrup should be less than 200 mm or the thickness of the jacketing section and less than 100 mm in the plastic hinge region of the column.
(4) The dust should be removed as the preparation of the concrete surface before jacketing.

**Figure 2:**
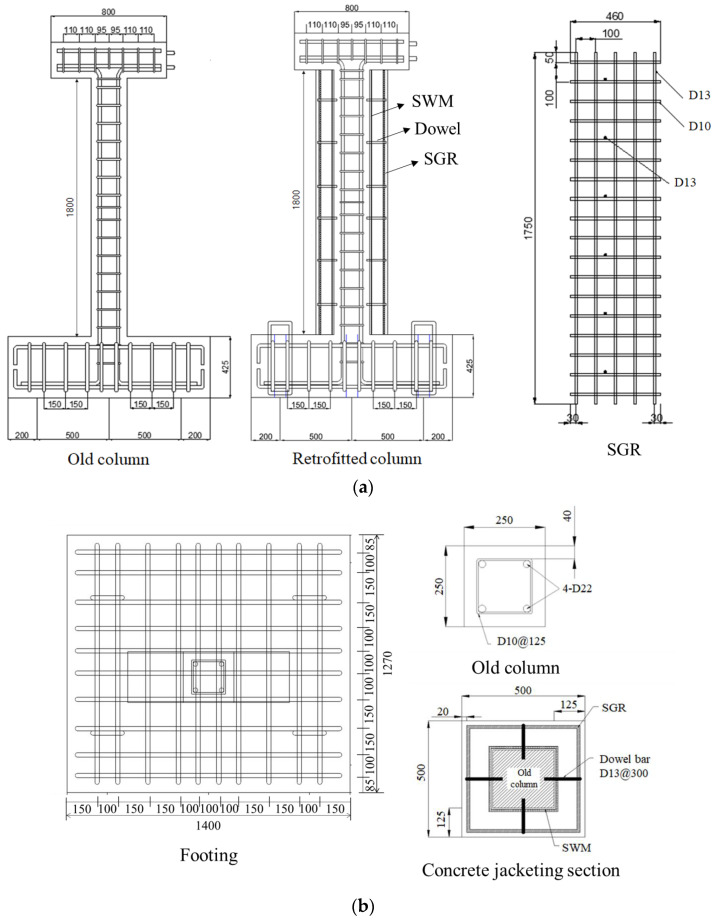
Specimen configuration (mm): (**a**) front view of specimens; (**b**) sections of members.

The sizes of retrofitted specimens were 500 mm × 500 mm, which had 250 mm thickness of the concrete jacketing section. Details of the specimens are shown in Figure 2 and listed in Table 1.

**Figure 3:**
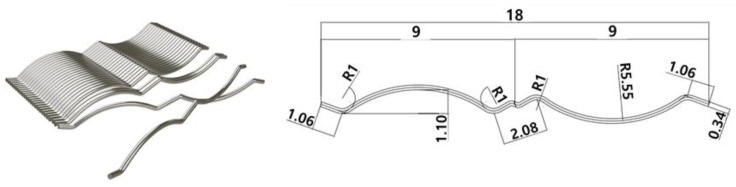
Steel fibers in SFNM (mm).

**Figure 4:**
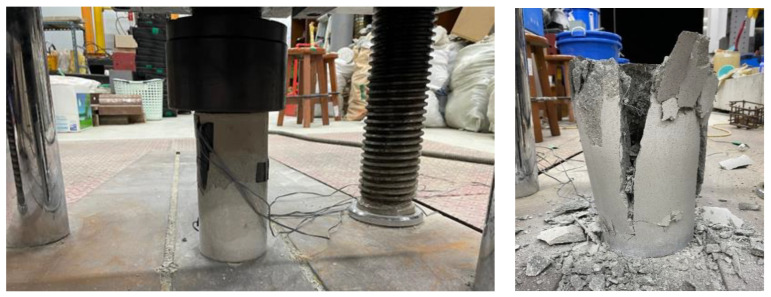
Uniaxial compressive test of SFNM.

Concrete with compressive strengths of 24 MPa was used for columns and upper beams. SFNM was used in the retrofitted specimens, which can be expected to control cracks caused by the shrinkage of concrete and coarse aggregates. Adding steel fibers can reduce the brittleness of concrete. The steel fiber content and shapes, such as straight, hooked-end, crimped, and twisted, affect its performance. Low fiber content (<1%) is generally used in plastic shrinkage crack control with minimal strength enhancement. Medium fiber content (1–2%) provides suitable workability for cast-in-place. High fiber content (>2%) can provide exceptional mechanical properties [17]. In addition, torsional resistance improves as the steel fiber content increases [18]. In this study, the hooked-end shape was used, which exhibited a better bond in the concrete mix and had better performance than the other types of fibers [17], and the steel fiber content was set at 1.5%, which is the most efficient content [19,20]. The steel fiber mixed in SFNM (Figure 3) is a roughened wire fiber with a double-arch shape glued in bundles. The details of steel fiber were same which used in previous study [15]. Double-arched steel fiber has a length of 18 mm and a diameter of 0.34 mm for an aspect ratio of 0.019. The maximum tensile strength of the fiber is 1250 MPa. The properties of steel fiber are listed in Table 2. The compressive strength of SFNM is 40.1 MPa, according to the results of a uniaxial compressive test, as shown in Figure 4. All specimens were designed in accordance with the specifications of the American Concrete Institute (ACI 318-19) [21]. Longitudinal reinforcement with a diameter of 22 mm was placed at the four corners. ACI 318-19 [21] recommended that the stirrup spacing should be less than the smallest value between 16 times the diameter of the longitudinal reinforcement, 48 times the diameter of the stirrup, and the smallest dimension of the member. According to Pennelis and Kappos [16], the spacing of the stirrup should be less than 200 mm or the thickness of the jacketing section. Therefore, D10 90-degree closed external stirrups were placed at 125 mm. The SWM and SGR consisted of steel rebar with respective diameters of 10 mm and 13 mm. The yield strength of the steel rebar in all specimens was 400 MPa.

### 2.2. Test Setup and Loading Protocol

**Figure 5:**
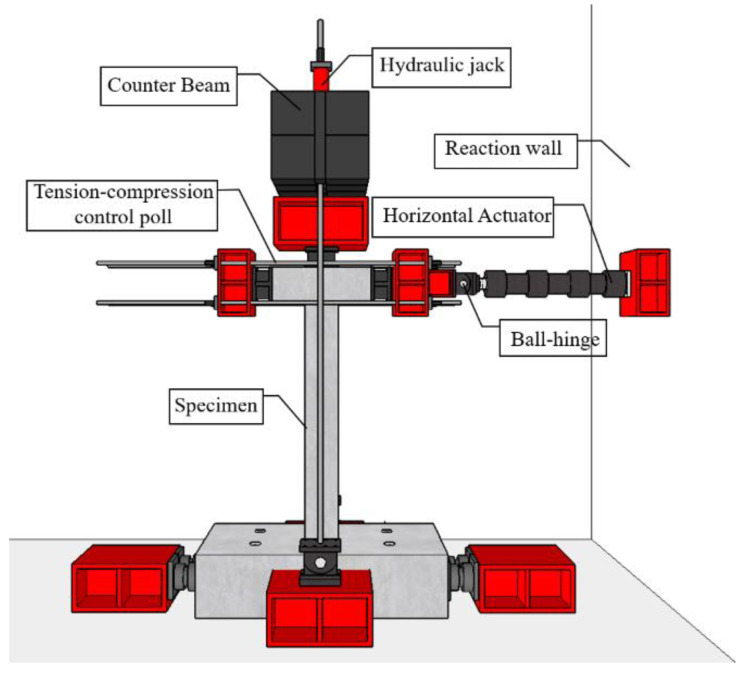
Test setup [15].

**Figure 6:**
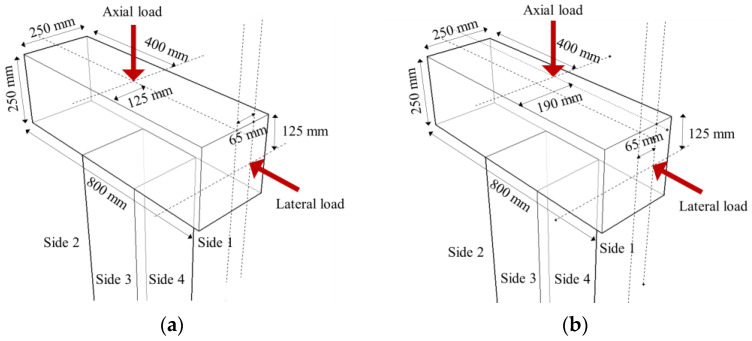
Loading scheme: (**a**) unidirectional, (**b**) bidirectional.

**Figure 7:**
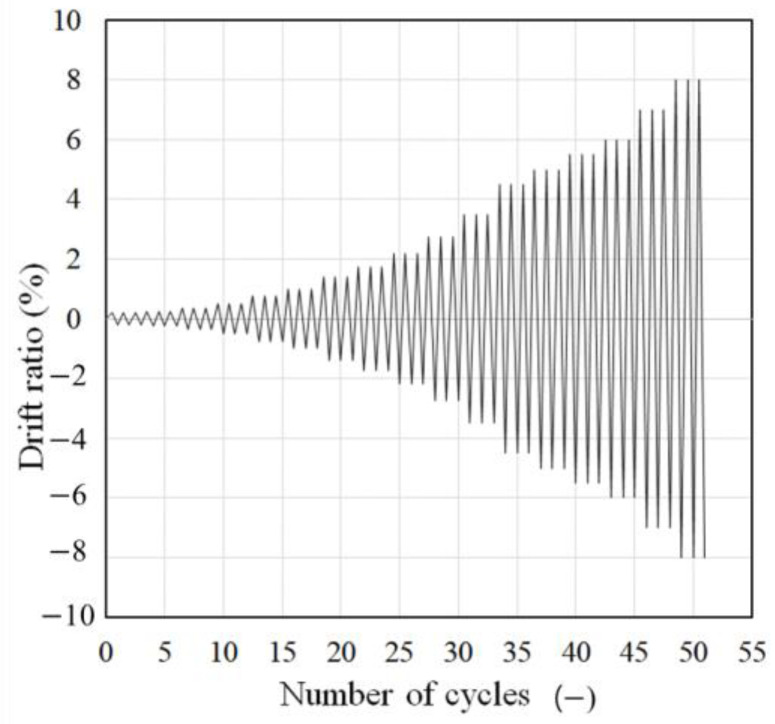
Loading protocol [15].

The detail of loading setup and loading protocol was referred to that of previous study [15]. Details of the loading setup are depicted in Figure 5 and Figure 6. The cyclic lateral load was applied by connecting the horizontal actuator and tension-compression control poll, which simulated actual earthquakes. To simulate a load condition similar to that of an actual building, a constant 255 kN compression, which was 17% of the axial load capacity, was applied to the upper beam using a hydraulic jack. To generate cyclic torsion, unidirectional and bidirectional loading were considered caused by eccentricity with respect to the load axis, as shown in Figure 6. These loading types generated eccentric lateral load, and only bidirectional loading generated eccentric axial load. In this study, the eccentric distance ($e_{1}$) of all specimens was set to 65 mm, which is 1/4 of the length of the section. In Figure 6, the front side of the specimen was designated as Side 1, and the elevations were divided by naming them Side 2, 3, and 4 in a counterclockwise direction to specify the direction. The quasi-static loading protocol considering actual seismic load was used, as shown in Figure 7. This loading was increased gradually from a drift ratio of 0.2% and was repeated three times at every step through a cyclic loading test method based on ACI 374.1-05 [22]. The drift ratio was calculated by dividing the lateral displacement by the length from the bottom of the column to the loading point, which was the loading point. There was no elapsed time between cycles.

## 3. Experimental Results

### 3.1. Cracks and Failure Patterns of Specimens

The cracks and failure patterns of each specimen at the end of the test are depicted in Table 3. Table 3 shows crack patterns in all directions from side 1 to side 4 of each specimen, in the same manner as in the previous study [15]. In this study, not only the lateral load but the torsion due to eccentricity was considered. Therefore, every specimen failed, showing complex crack patterns. In the case of the non-retrofitted specimen NSU, to which unidirectional loading was applied, the initial cracks occurred in the bottom of the column at a drift ratio of 1%. As the torsional load increased, shear cracks developed in the center of the column at a drift ratio of 2.2%. At a drift ratio of 2.75%, the load reached the maximum, the width of shear cracks increased significantly, and concrete spalling was observed at the bottom of the column. The experiment was terminated at a drift ratio of 4.5%, occurring severe cracks and concrete crushing. In the case of non-retrofitted specimen NSB, to which bidirectional loading was applied, the initial cracks occurred at a drift ratio of 0.75%, and as the torsional load increased, shear cracks occurred at a drift ratio of 1.4%. Thereafter, the increase in the number and width of cracks was insignificant compared to that of NSU. After reaching a maximum load at a drift ratio of 3.5%, concrete spalling occurred at the bottom of the column. The experiment was terminated due to concrete crushing occurring in the column-foundation joint at a drift ratio of 5.5%. These results indicated that flexural and torsional moments generated by combined load decreased as the axis of the lateral and axial loads of the bidirectional loading coincided. In the case of SFU and SFB, the initial cracks occurred at a drift ratio of 0.2%. The shear cracks occurred at a drift ratio of 1% for SFU and 0.75% for SFB. After that, no critical cracks or increases in the width of existing cracks were observed. Concrete spalling occurred in the bottom of the column at drift ratios of 3.5% and 2.75% for SFU and SFB, respectively. The experiment was terminated due to observing failure in the bottom of the column at a drift ratio of 8.5% for SFU and 6% for SFB. The number and width of shear cracks were significant in SFU compared with SFB. In addition, the failure in column-foundation joints in SFU was more severe than that of SFB. It was the same reason as that of the non-retrofitted specimens. If the specimens with a low ratio of torsion moment to the bending moment (T/M), flexure-dominant behavior is observed. When T/M ratio is higher, more diagonal cracks appear, and the angle becomes larger. It is indicated that the failure type changed from flexural failure to torsional failure because the typical torsion failure is accompanied by diagonal cracks at a 45-degree angle [23]. For the specimens subjected to unidirectional load, T/M ratio was higher than that of bidirectional load. Therefore, more diagonal cracks were observed and gradually developed upward in the specimens under unidirectional load.

The comparison of crack propagation between the non-retrofitted column and the concrete-jacketed column is summarized in Table 4. The pictures in Table 4 are the bottoms of the columns at which significant cracks can be observed. From Table 4, it can be recognized that the damage to the retrofitted column is much less in the same state.

In the retrofitted specimens using the hybrid concrete jacketing method, fewer cracks occur than in the non-retrofitted specimens, regardless of the loading scheme. It is indicated that the hybrid concrete jacketing method can effectively resist torsion and shear.

### 3.2. Load-Displacement Relationships

**Figure 8:**
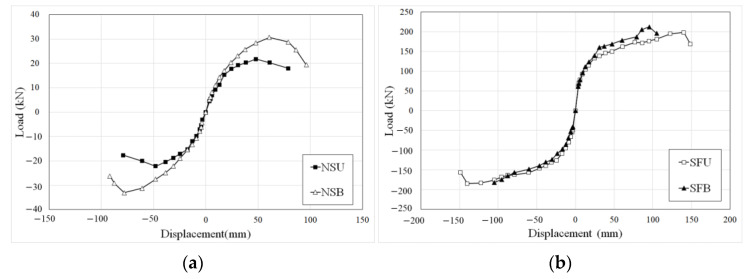

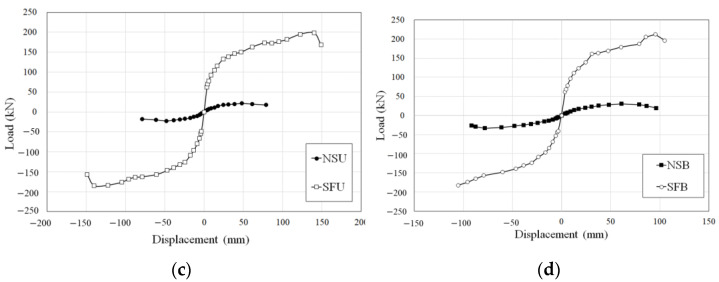
Skeleton curves: (**a**) NSU and NSB, (**b**) SFU and SFB, (**c**) NSU and SFU, and (**d**) NSB and SFB.

To compare the maximum load and drift ratio for each specimen, the skeleton curves are shown in Figure 8. The maximum loads and displacements of all specimens are listed in Table 5. The maximum load and maximum displacement of NSU were approximately 34% and 18% of that of NSB, respectively. As applying bidirectional loading, in-plane torsion due to lateral loads and out-of-plane torsion due to axial loads act on the same axis of load decreased torsional moment. In the case of the specimen in which unidirectional loading is applied, because the axes of the lateral and axial loads did not coincide, the in-plane torsion caused by the lateral load affected the failure of the specimen. The maximum loads of SFU and SFB were recorded as 198.32 kN and 226.22 kN, respectively, and the maximum displacements were 148.76 mm and 105.06 mm, respectively. The maximum load of SFB was 1.14 times that of SFU. The maximum displacement of SFU was 1.41 times that of SFB. More torsion was generated in SFU because axial load and lateral load did not apply coincidence, and eccentric axial load in SFB generated out-of-plane torsion. Therefore, in the case of SFB, which applied bidirectional load, the maximum load increased, although the maximum displacement decreased that of SFU.

The maximum load and displacement of SFU were 9.2 times and 1.9 times that of NSU, respectively. In addition, the maximum load and displacement of SFB were 7.4 times and 1.1 times that of NSB, respectively. There was no dramatic reduction in the retrofitted specimens in any of the loading schemes because the hybrid concrete jacketing method can effectively resist improving the seismic performance of the old columns.

### 3.3. Torsional Moment Versus Twist Response

A cyclic loading test was conducted considering the combination of bending, shear, and axial load, which can generate torsion due to structural irregularity. The effect of the applied loading scheme on the column and the performance of the retrofit method were analyzed in terms of the relationship between the torsional moment and the twist. The torsional moment and twist are calculated by Equations (1) and (2). The torsional moment and twist were obtained from LVDTs and strain gauges.

(1) $$M_{i}=P\times \ell \times {\cos\theta }_{i}$$

(2) $$\theta_{i}=\tan^{-1}\left(\frac{\Delta_{2}-\Delta_{1}}{d}\right)$$

**Figure 9:**
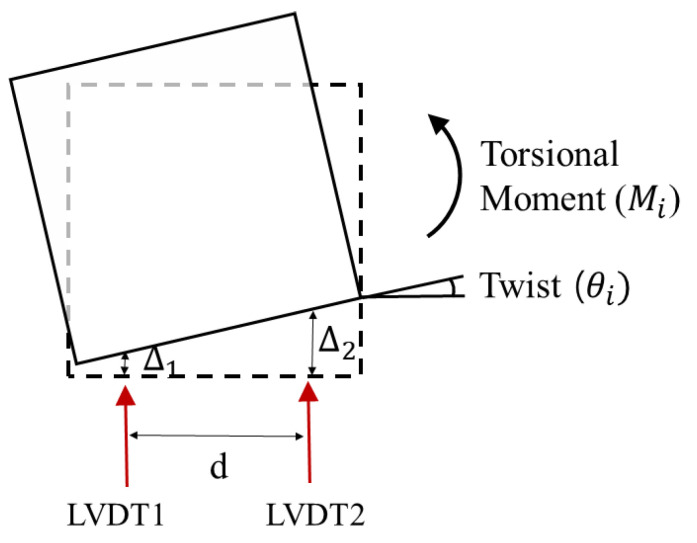
Torsional moment and twist of columns.

Here, $M_{i}$ is the torsional moment generated to the specimen at the i-th drift ratio, P is the maximum load at the i-th drift ratio, ℓ is the eccentric distance, and $\theta_{i}$ is the twist of the column cross-section, calculated as shown in Figure 9.

**Figure 10:**
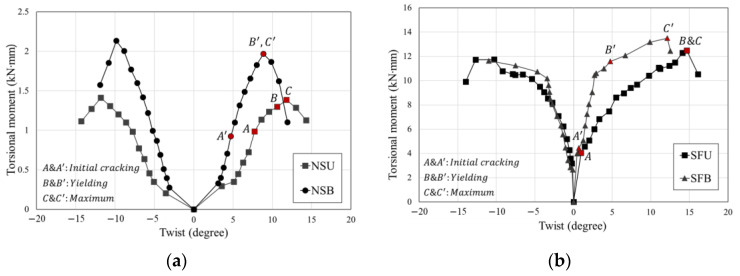
Torsional moment-twist envelopes: (**a**) NSU and NSB and (**b**) SFU and SFB.

All torsional moments were calculated using the first cycle, which recorded the maximum load of that drift ratio. The torsional moment-twist envelopes are shown in Figure 10. When the T/M ratio is lower, horizontal cracks are exhibited, and as T/M ratio increases, diagonal cracks appear, as shown in Table 4. The specimens failed due to extending existing cracks. The increase in the T/M ratio indicated an increase in the torsional moment under the same loading scheme. Therefore, the applied torsional moment and twist at concrete cracking, longitudinal reinforcement yielding, and maximum torsional moment and twist for old and retrofitted columns are analyzed in Table 6. At the beginning of testing all specimens, the response was linear with increasing applied torsional moment until the initial crack occurred. The torsional moments of non-retrofitted specimens NSU and NSB reached 0.98 kN·m and 0.92 kN·m, respectively. The torsional moment of SFU was 4.1 times that of NSU, and also, in the case of SFB, the torsional moment was 4.8 times that of NSB. In the case of NSU and NSB, the increasing rates of the torsional moment from the initial cracking to the yielding were 25% and 53%, respectively. In addition, the increasing rates of SFU and SFB were 68% and 62%, respectively. The maximum torsional moment had a similar value at longitudinal reinforcement yielding. The phenomenon that the retrofitted specimens were able to provide more torsional resistance can be explained by improved confinement using the hybrid concrete jacketing method.

### 3.4. Torsional Stiffness

**Figure 11:**
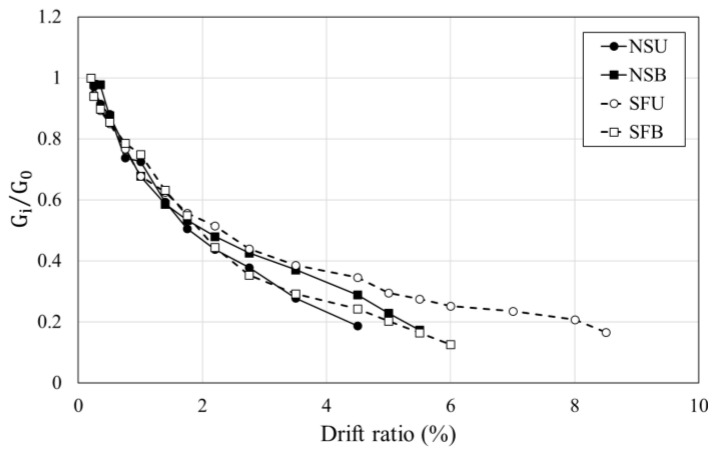
Torsional stiffness degradation of specimens.

The concrete jacketing method has advantages for improving the strength and stiffness of old columns. He et al. [24] expressed the torsional stiffness attenuation using the relationship between $G_{i}$/$G_{0}$ and twist. Here, $G_{i}$ denotes the torsional stiffness at the i-th drift ratio and $G_{0}$ denotes the initial torsional stiffness determined from the first loading cycle. Figure 11 shows the torsional stiffness attenuation according to the increased drift ratio. The initial torsional stiffness of NSU and NSB was 1.2 kN/mm and 1.4 kN/mm, respectively. The hybrid concrete jacketing method improved the initial torsional stiffness of the RC column subjected to unidirectional load 12 times, and in the case of specimens subjected to unidirectional load, retrofitted specimens were 10 times that of the non-retrofitted column. The retrofitted columns using the hybrid concrete jacketing method exhibited a similar trend in torsional stiffness attenuation as the non-retrofitted columns at the beginning of the experiment. With further increases in applied loading and shear crack occurring, in the case of SFU and SFB, the torsional stiffness decreased slower than NSU and NSB. The torsional stiffness of the non-retrofitted specimen rapidly decreased after the maximum torsional moment was reached due to concrete spalling. SFB had lower initial stiffness against torsion compared with that of SFU, but there was little difference in the torsional stiffness degradation. As T/M ratio increased, significant shear cracks occurred, and the concrete cover started spalling, which indicated significant torsional stiffness degradation. The torsional stiffness decreased after observation of the initial crack in all specimens. Although the T/M ratio in unidirectional load is larger than that in bidirectional load, it was confirmed that the rate of decrease in torsional stiffness of SFU was similar to that of SFB. This confirmed that the hybrid concrete jacketing method showed constant torsional resistance even under various loading schemes.

### 3.5. Energy Dissipation

**Figure 12:**
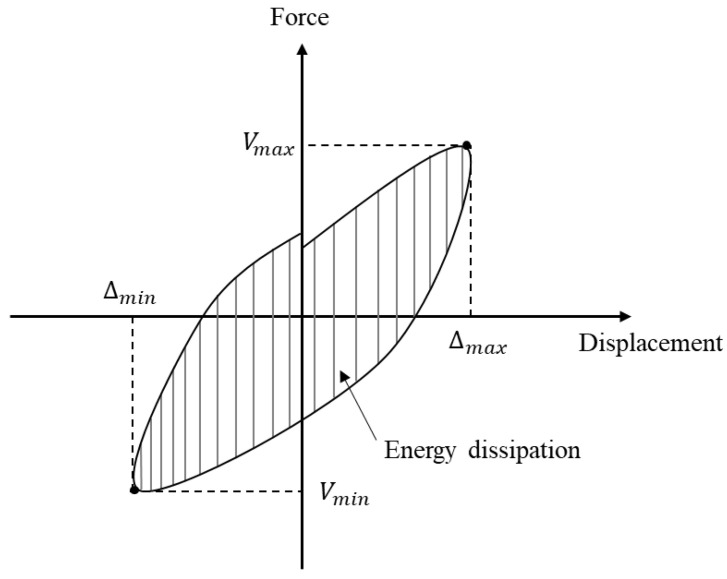
Definition of energy dissipation.

Energy dissipation capacity is one of the most important indicators to evaluate the seismic performance of structures. Energy dissipation was typically estimated by calculating the area enclosed by the hysteresis loop for each cycle, as shown in Figure 12 [25,26]. In this paper, the energy dissipation was calculated using the maximum values in the first cycle.

**Figure 13:**
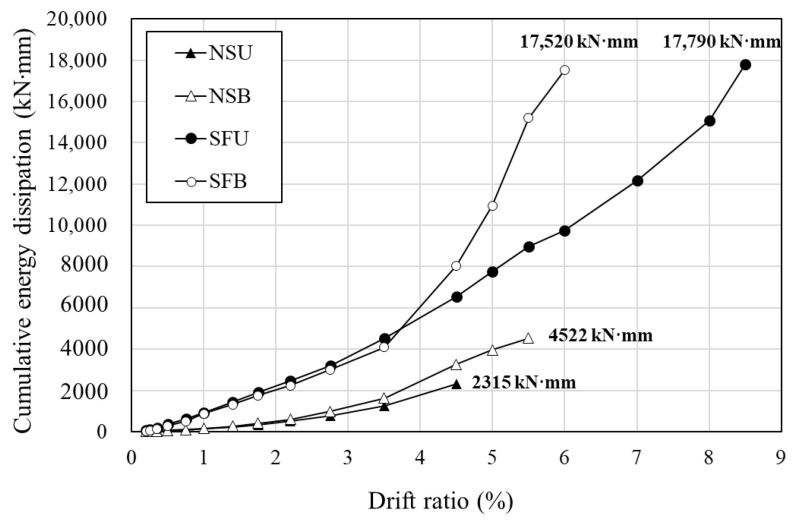
Comparison of cumulative energy dissipation.

The comparison of the cumulative energy dissipation capacity of each specimen is shown in Figure 13. The cumulative energy dissipation at the ultimate point of NSU, NSB, SFU, and SFB was 2315 kN·mm, 4522 kN·mm, 17,790 kN·mm, 17,520 kN·mm, respectively. At the point of failure of NSU, which has a drift ratio of 4.5%, the cumulative energy dissipation of NSB was 1.4 times that of NSU. The cumulative energy dissipation capacity of SFB until a drift ratio of 6% when SFB failed was approximately 1.8 times that of SFU. Concrete-jacketed columns dissipate much more energy than the old columns in general. This is because the concrete jacketing method can improve strength and stiffness by enlarging the cross-section [27,28]. As shown in Figure 13, the hybrid concrete jacketing method improved the energy dissipation capacity of the old column. In addition, it was confirmed that the hybrid concrete jacketing method, in unidirectional loading, in which more torsion is induced, can enhance energy dissipation capacity much more than that in bidirectional loading. It was indicated that the hybrid concrete jacketing method secures the bond and confinement between the existing member and the jacketed section and can also resist the torsional load.

## 4. Conclusions

This study investigated the torsional behavior of retrofitted RC columns using the hybrid concrete jacketing method under combined loading. Two non-retrofitted RC column specimens and two retrofitted specimens were fabricated, and cyclic loading tests were conducted. Two types of loading schemes were applied to simulate actual seismic load. The following conclusions have been drawn:

(1) In the case of specimens under unidirectional loading, more significant shear cracks and concrete spalling occurred than that in specimens under bidirectional loading. These results indicated that flexural and torsional moments generated by combined load decreased as the axes of the lateral and axial loads of the bidirectional loading coincided. In the retrofitted specimens using the hybrid concrete jacketing method, fewer cracks occurred than in the non-retrofitted specimens, regardless loading scheme. It is indicated that the hybrid concrete jacketing method can effectively resist torsion and shear.
(2) The maximum load of SFU was about 9.2 times larger than NSU, and that of SFB was 7.4 times larger than NSB. In addition, the hybrid concrete jacketing method increased the cumulative energy dissipation capacity by approximately 7.7 times compared to the old RC columns under unidirectional loading, in which more torsion is induced. Therefore, the hybrid concrete jacketing method can effectively provide torsional resistance without the complex retrofitting process in the existing concrete jacketing method.
(3) For all specimens, the response was linear with increasing applied torsional moment until the initial crack occurred. The torsional moment of SFU was about 4.1 times larger than NSU, and that of SFB was 4.8 times larger than NSB. In the case of NSU and NSB, the increasing rates of the torsional moment from the initial cracking to the maximum torsional moment were 30% and 53%, respectively, and that of SFU and SFB were 68% and 67%, respectively. For torsional stiffness attenuation, the hybrid concrete jacketing method improved the initial torsional stiffness of the RC column subjected to unidirectional load 12 times, and in the case of specimens subjected to unidirectional load, retrofitted specimens were 10 times that of the non-retrofitted column. The torsional stiffness of the non-retrofitted specimens rapidly decreased after the maximum torsional moment was reached due to concrete spalling. This indicated that the hybrid concrete jacketing method could significantly enhance the torsional capacity.

## Figures and Tables

**Table 1:** Details of specimens.

Specimen	Retrofit Method	Loading Scheme	Cross Section (mm)	Materials	
				Concrete (MPa)	Steel (MPa)
NSU	–	Unidirectional	250 × 250	24	400
NSB		Bidirectional			
SFU	Concrete jacketing	Unidirectional	500 × 500		
SFB		Bidirectional			

**Table 2:** Geometrical and mechanical properties of steel fiber [15].

Material	Diameter (mm)	Length (mm)	Aspect Ratio	Tensile Strength (MPa)
Low carbon	0.34	18	0.019	1250

**Table 3:** Crack patterns of specimens at failure.

Specimens	Side 1	Side 2	Side 3	Side 4
NSU	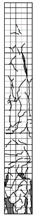	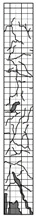	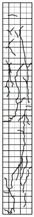	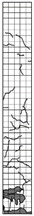
NSB	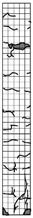	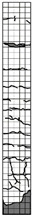	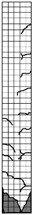	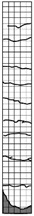
SFU	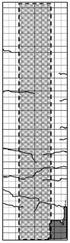	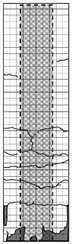	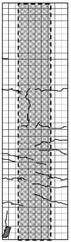	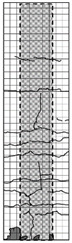
SFB	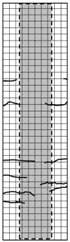	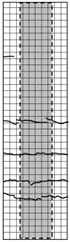	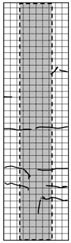	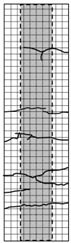

**Table 4:** Crack propagation.

State	NSB		SFB	
	Drift Ratio (%)	Photograph	Drift Ratio (%)	Photograph
Initial shear crack	1.4	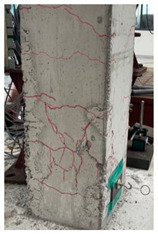	1.75	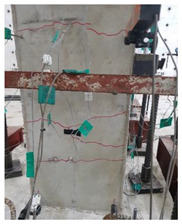
Concrete spalling	3.5	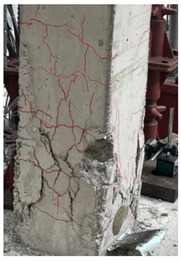	2.75	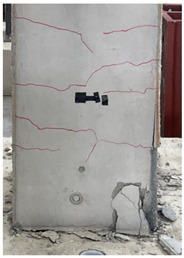
Failure	5.5	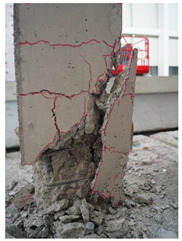	6	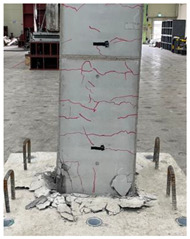

**Table 5:** Maximum load and displacement from test results.

Specimen	Maximum Load (kN)		Maximum Displacement (mm)	
	Positive (+)	Negative (−)	Positive (+)	Negative (−)
NSU	21.61	−22.26	79.08	−79.03
NSB	30.66	−33.32	96.35	−96.38
SFU	198.32	−184.75	148.76	−148.75
SFB	226.22	−182.29	105.06	−105.03

**Table 6:** Comparison of torsional performance of specimens.

		NSU		NSB		SFU		SFB	
		(+)	(−)	(+)	(−)	(+)	(−)	(+)	(−)
Cracking	$M_{T,cr}$ (kN·m)	0.98	0.98	0.92	0.87	4.03	3.18	4.45	2.84
	$\theta_{T,cr}$ (degree)	7.75	7.75	4.73	4.71	0.96	0.92	0.68	0.56
Yield	$M_{T,y}$ (kN·m)	1.30	1.31	1.97	2.00	12.47	11.72	11.59	9.62
	$\theta_{T,y}$ (degree)	10.64	10.64	8.88	8.87	14.67	12.69	4.76	3.24
Maximum	$M_{T,max}$ (kN·m)	1.38	1.41	1.97	2.13	12.47	11.87	13.50	11.63
	$\theta_{T,max}$ (degree)	14.30	14.30	11.92	11.92	14.66	8.75	12.16	10.97

## Data Availability

Not applicable.
